# Negative pressure wound therapy in the setting of acute abdominal evisceration secondary to major thoracoabdominal burns

**DOI:** 10.1093/jscr/rjaf203

**Published:** 2025-04-08

**Authors:** William T Crawley, Benson Pulikkottil, Maxwell Busch

**Affiliations:** Department of Graduate Medical Education, HCA HealthOne - Swedish Medical Center, 610 E Hampden, Ste 220, Englewood, CO 80113, United States; HCA HealthOne - Swedish Medical Center, Burn and Reconstructive Center, 610 E Hampden, Ste 310, Englewood, CO 80113, United States; The LaVie Institute, 10535 Park Meadows Blvd. #350, Lone Tree, CO 80124, United States; Department of Trauma Surgery, HCA HealthOne - Swedish Medical Center, 610 E Hampden, Ste 220, Englewood, CO 80113, United States

**Keywords:** thoracoabdominal burns, negative pressure wound therapy, temporary abdominal closure, trauma, exploratory laparotomy

## Abstract

Negative pressure wound therapy has allowed for improved management of critical ill patients who requiring abdominal exploration by minimizing operation length, expediting resuscitation, and avoiding unnecessary resections. The use of this vital resource is often limited in burn patients with involvement of the thoracoabdominal wall due to difficulty with maintaining a seal necessary for the negative pressure. This report highlights the case of a 57-year-old patient who sustained significant thoracoabdominal burns and suffered an acute evisceration of small bowel following a debridement. The patient required an emergent return to the operating room and temporary abdominal closure in order to allow for a second look laparotomy. We detail the use of ostomy/barrier rings in order to establish a seal necessary for the negative pressure wound therapy. This novel technique has the potential to expand the use of temporary abdominal closure using negative pressure wound therapy in clinically injured burn patients.

## Introduction

The development of negative pressure wound therapy (NPWT) or wound vacs has revolutionized the world of medicine, especially as it relates to the management options for critically ill patients with intra-abdominal pathology [1–4]. As the body of research regarding damage control surgery has grown, focus has shifted to minimizing time in the operating room (OR) for index operations [5–7]. This allows for faster stabilization and resuscitation, factors that are foundational in critically injured burn patients [8], as well as potentially minimizing unnecessary intra-abdominal resections through second-look explorations [9]. This is where NPWT has become vital, as it allows for temporary abdominal closure while providing fluid removal, reduction in edema and lateral wall retraction, and infection control [10]. One of the major difficulties with abdominal placement of wound vacs is related to achieving an adequate occlusive seal necessary for generating the negative-pressure, something that can be especially challenging in patients with abdominal trauma and burn injuries. This case highlights the successful use of a wound vac in a patient with significant thoracoabdominal burn injuries who required laparotomy secondary to acute abdominal evisceration.

## Case presentation

Our case is a 57-year-old male who was admitted to the burn intensive care unit at a level I trauma center following a trailer home explosion in which the patient sustained major burns totaling 56% total body surface area. Full thickness burns involved the bilateral feet and the majority of the patient’s anterior thorax and abdomen. Partial thickness burns were located over the bilateral upper extremities and the facial region. The patient also sustained significant smoke inhalation which necessitated intubation on arrival. No other traumatic injuries were noted at admission.

**Figure 1:**
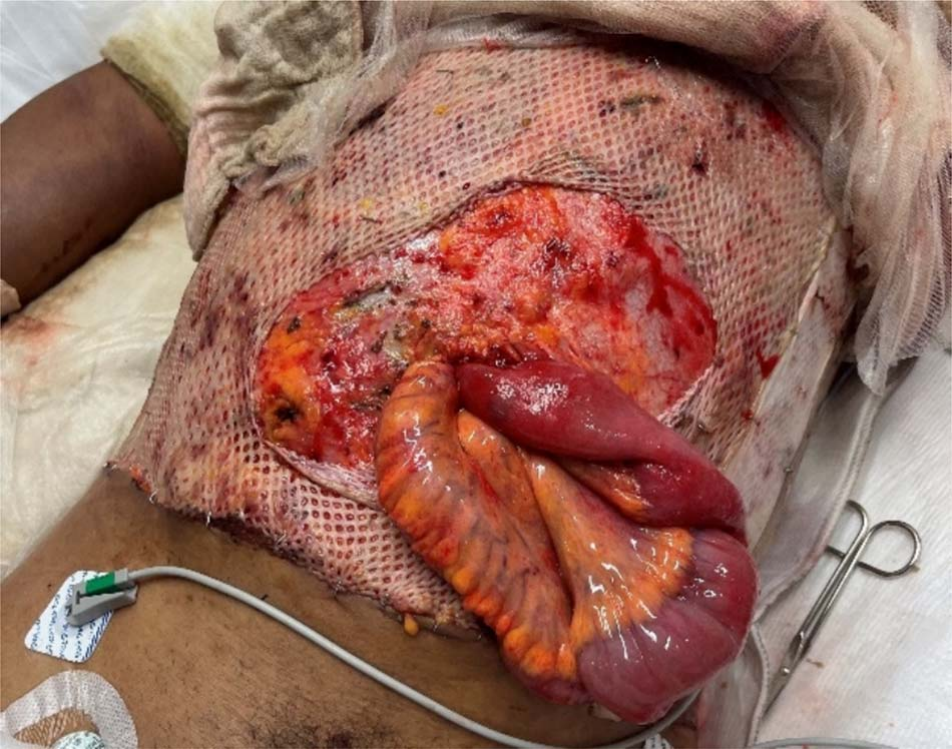
Acute evisceration of small bowel noted in the burn intensive care unit following an episode of coughing secondary to ventilator dyssynchrony.

Due to the extent of the patient’s injuries, he required multiple trips to the OR for repeated excisional debridement including deep facial debridement over the thorax and abdomen. During one of the debridements, the patient was noted to have a small fascial defect in the umbilical region that was not believed to communicate with the peritoneum. The defect was closed with PDS suture and allograft placed over the defect. Post operatively, patient had increased abdominal pressure due to ventilator dyssynchrony and significant coughing. The following morning, nursing staff reported increased mid-abdominal distention below the burn dressings. When the dressings were removed, the patient was noted to have evisceration of a portion of small bowel (Fig. 1). The small bowel was reduced at the bedside and the patient was then taken for an emergent exploratory laparotomy.

**Figure 2:**
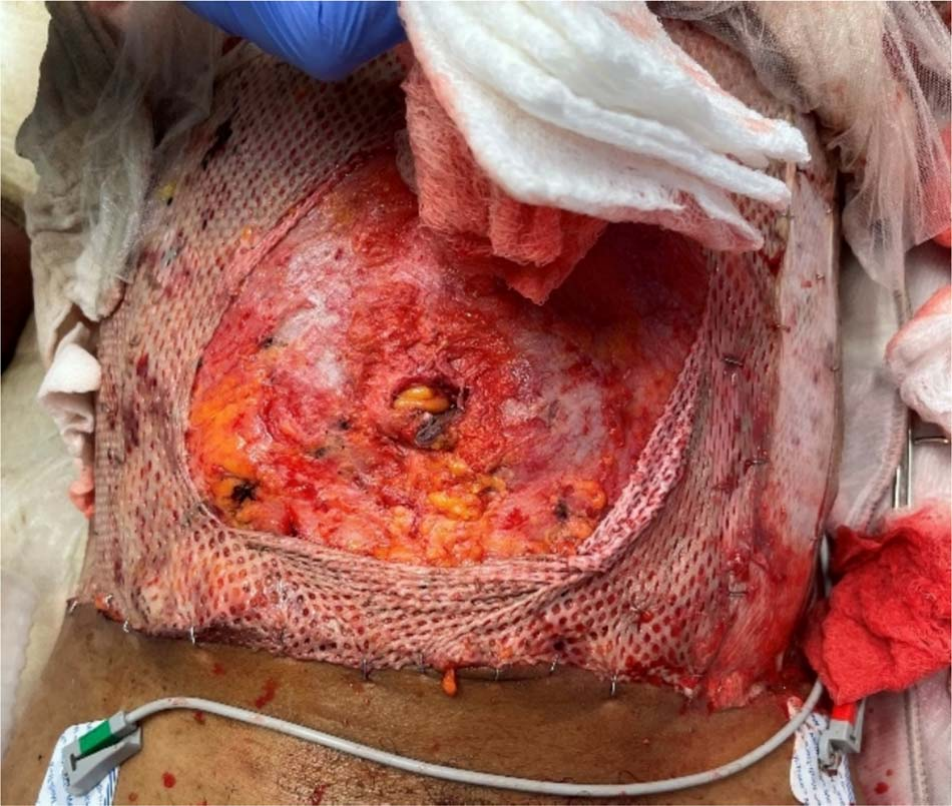
1.8 cm supraumbilical fascial defect noted in the operating room following reduction of the eviscerated small bowel.

**Figure 3:**
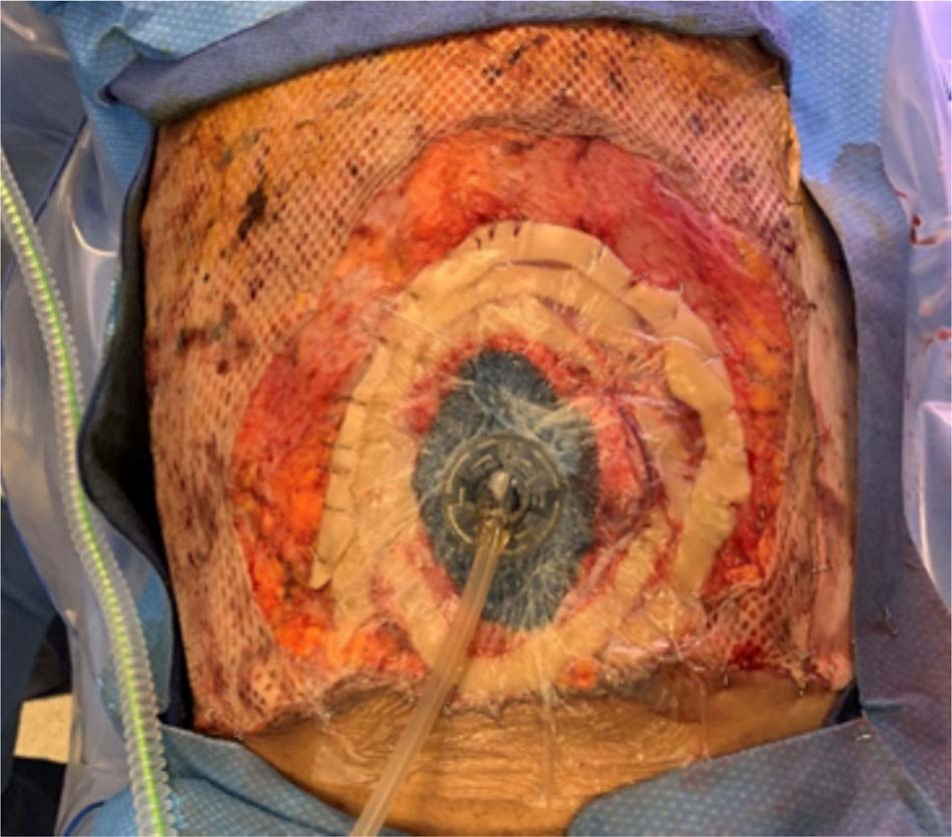
Concentrically applied ostomy/barrier rings that were secured to the anterior abdominal wall fascia in order to allow for sealing of the wound vac.

In the OR, a 1.8 cm supraumbilical fascial defect was noted (Fig. 2), which was extended in the cephalad and caudal directions to allow for adequate exposure. Upon examination of the bowel, a mesenteric hematoma of the terminal ileum was noted along with multiple areas of ecchymosis involving the ileal wall. There was no evidence of necrosis or ongoing ischemia warranting immediate resection. Due to the patient’s critical status and concern for bowel viability, the decision was made for temporary abdominal closure using a wound vac and plan for a second-look laparotomy in 24–48 h. Given the patient’s significant abdominal burns and extensive tissue debridement, securing the wound vac was a unique challenge. This was achieved with the use of ostomy/barrier rings placed in a concentric fashion around the defect and secured with staples to the anterior abdominal wall fascia. This allowed for adequate adhesion of the occlusive dressing necessary for NPWT function (Fig. 3).

A total of 24 h later, the patient was taken back to the OR for a second-look laparotomy with all of the bowel appearing viable. The anterior abdominal wall fascial defect was then closed using 0 looped PDS suture in a running fashion and supplemental 0 Vicryl retention sutures in a figure of eight fashion. The defect was then covered with allograft by the burn surgery team.

## Discussion

Temporary abdominal closure in critically ill patients is beneficial because it minimizes OR time, prioritizes resuscitation, and allows for repeated abdominal examination of borderline organ viability; therefore, potentially avoiding unnecessary resections [9]. This is especially important in burn patients who require extensive fluid resuscitation and can often have associated traumatic thoracoabdominal injuries requiring surgical exploration [11]. In these patients, the use of abdominal wound vacs provides the benefit of removing intraperitoneal fluid and reducing the risk of secondary infection, minimizing lateral retraction of the abdominal wall, and allowing for repeated examination of the abdomen. While the abdominal wound vac has tremendous benefit, its use in burn patients with involvement of the anterior abdominal wall has been limited due to difficulty with application of the wound vac and obtaining a seal. This case highlights the successful use of an abdominal wound vac in a patient with major thoracoabdominal burns using layered ostomy/barrier rings to seal the wound vac. Further randomized controlled studies are needed to completely identify the risks and benefits of this novel approach, but, to our knowledge, this is one of the first cases to demonstrate such a technique. We believe that the use of ostomy/barrier rings will allow for enhanced and more frequent use of NPWT in burn patients requiring temporary abdominal closure, therefore reducing the risk of abdominal sepsis, lateral abdominal wall retraction, and unnecessary organ resections.
